# Comparison of clinical outcomes and quality of life for robotic versus laparoscopic surgery in elderly patients with mid-low rectal cancer: a multicenter cohort study with inverse probability of treatment weighting analysis

**DOI:** 10.3389/fonc.2026.1809161

**Published:** 2026-04-21

**Authors:** Lei Zhang, Kexuan Wang, Guanghui Wang, Chenhao Hu, Chao Yin, Yujie Li, Lin Wang, Junjun She, Zhigang Wu, Feiyu Shi

**Affiliations:** 1 Yulin Hospital, The First Affiliated Hospital of Xi'an Jiaotong University, Yulin, Shaanxi, China; 2 Department of General Surgery of Yulin Hospital, The First Affiliated Hospital of Xi'an Jiaotong University, Yulin, Shaanxi, China; 3 Department of General Surgery, The First Affiliated Hospital, Xi'an Jiaotong University, Xi’an, Shaanxi, China

**Keywords:** mid-low rectal cancer, elderly population, robotic surgery, inverse probability of treatment weighting, quality of life

## Abstract

**Background:**

The rising incidence of rectal cancer (RC) in elderly patients contrasts with the scarcity of robust evidence comparing robotic-assisted and laparoscopic resection in this population. This study evaluated clinical outcomes and quality-of-life (QoL) metrics between these approaches in elderly patients with mid-low RC.

**Methods:**

Consecutive patients aged 70 years or older with mid-low RC who underwent minimally invasive resection at two tertiary centers from January 2017 to December 2024 were categorized into robotic (n=211) and laparoscopic (n=812) cohorts. Outcomes were analyzed using inverse probability of treatment weighting (IPTW) based on propensity scores.

**Results:**

After IPTW, the effective sample comprised 2036 patients (1012 robotic group, 1024 laparoscopic group) with balanced clinicopathological characteristics. After matching, the pure operation time was comparable between groups (*P*>0.05), but the robotic approach achieved superior intraoperative outcomes, with significantly reduced estimated blood loss and lower incidence of intraoperative complications (all *P* < 0.05). Postoperatively, the cohort undergoing robotic-assisted procedures exhibited significantly lower rates of both overall complications and specific incidents of urinary retention. Longitudinal QoL assessment revealed sustained advantages in functional recovery, with the robotic cohort showing significantly better preserved urinary function, defecatory function, and male sexual function throughout the 12-month follow-up period (all *P* < 0.05). For patients with T4 stage tumors, the robotic approach demonstrated superior cancer-specific survival compared to laparoscopy, a benefit that was maintained after IPTW.

**Conclusions:**

Robotic-assisted surgery is a safe and feasible alternative for elderly patients with mid-low RC, offering reduced perioperative complications, superior functional preservation, and potential CSS benefits in T4 disease.

## Introduction

Globally, occurring predominantly in elderly patients, rectal cancer (RC) accounted for an estimated 729,702 new diagnoses and 343,776 fatalities in 2022 (1). Given rapidly aging populations and extended life expectancies, the number of elderly RC patients undergoing surgical intervention—the gold-standard curative therapy—is projected to rise continuously. Compared with young patients, elderly patients have significantly higher postoperative morbidity and mortality due to decreased immunity, underlying diseases, organ dysfunction, cognitive dysfunction and surgical stress-induced circulatory and respiratory system abnormalities (2, 3). In addition, due to deficient screening implementation and limited awareness of early detection strategies, elderly patients with RC often present with advanced disease at initial diagnosis (4). Advanced tumor stages correlate with deeper tissue invasion and more extensive peritumoral adhesions, consequently elevating both surgical complexity and perioperative complication risks. Thus, surgical treatment of mid-low RC in elderly patients presents serious challenges to surgeons and places heavy medical burdens on patients and the healthcare system.

The evolution of RC surgery has witnessed significant technical advancements. Since its introduction, total mesorectal excision (TME) has become the widely accepted standard for mid-low RC resection (5). While laparoscopic TME demonstrates clear perioperative advantages compared to open procedures, with robust evidence supporting comparable long-term oncological results (6–9), this approach presents inherent technical constraints. The non-articulating design of conventional laparoscopic instruments creates operative challenges in the restricted pelvic cavity, often resulting in spatial instrument interference and suboptimal ergonomic positioning for surgeons. Furthermore, inherent limitations including two-dimensional visualization, instrument-hand incongruence, and substantial proficiency requirements remain unresolved issues in laparoscopic TME procedures. Subsequently, the introduction of robotic surgical technology has enabled a minimally invasive approach that addresses the technical limitations associated with laparoscopic TME, thereby enhancing the feasibility of performing intricate pelvic operations. Its advantages, including stereoscopic visualization, articulating instrument arms, and tremor-free camera stabilization, enable precise tumor resection while preserving critical pelvic neurovascular structures. The landmark REAL trial demonstrated superior oncological resection quality in robotic-assisted TME for mid-low RC compared to conventional laparoscopy, along with reduced surgical invasiveness and enhanced postoperative recovery (10). However, subsequent multicenter randomized control trials (RCTs) [COLRAR (11) and ROLARR (12)] revealed no statistically significant disparities in either TME specimen quality or open conversion rates between robotic and laparoscopic approaches. Current evidence consistently supports the efficacy and safety of robotic TME in appropriately selected patients.

Elderly patients with RC present clinical challenges due to concurrent physiological decline, psychosocial vulnerabilities, and comorbidities that collectively elevate risks for adverse clinical outcomes (13, 14). This inherent heterogeneity has led to systematic exclusion of geriatric populations in most rigorous clinical trials through stringent eligibility criteria. Notably, robust multicenter comparisons evaluating robotic versus laparoscopic approaches in elderly RC patients remain conspicuously absent in contemporary literature. The exact role of this promising robotic technology in this vulnerable demographic is still unclear.

Therefore, this study was structured to perform a comparative evaluation of perioperative results, long-term survival rates, and postoperative quality of life (QoL) among older adults with mid-low RC, aiming to evaluate the practicality and effectiveness of robot-assisted versus laparoscopic techniques.

## Materials and methods

### Study population

**Figure 1:**
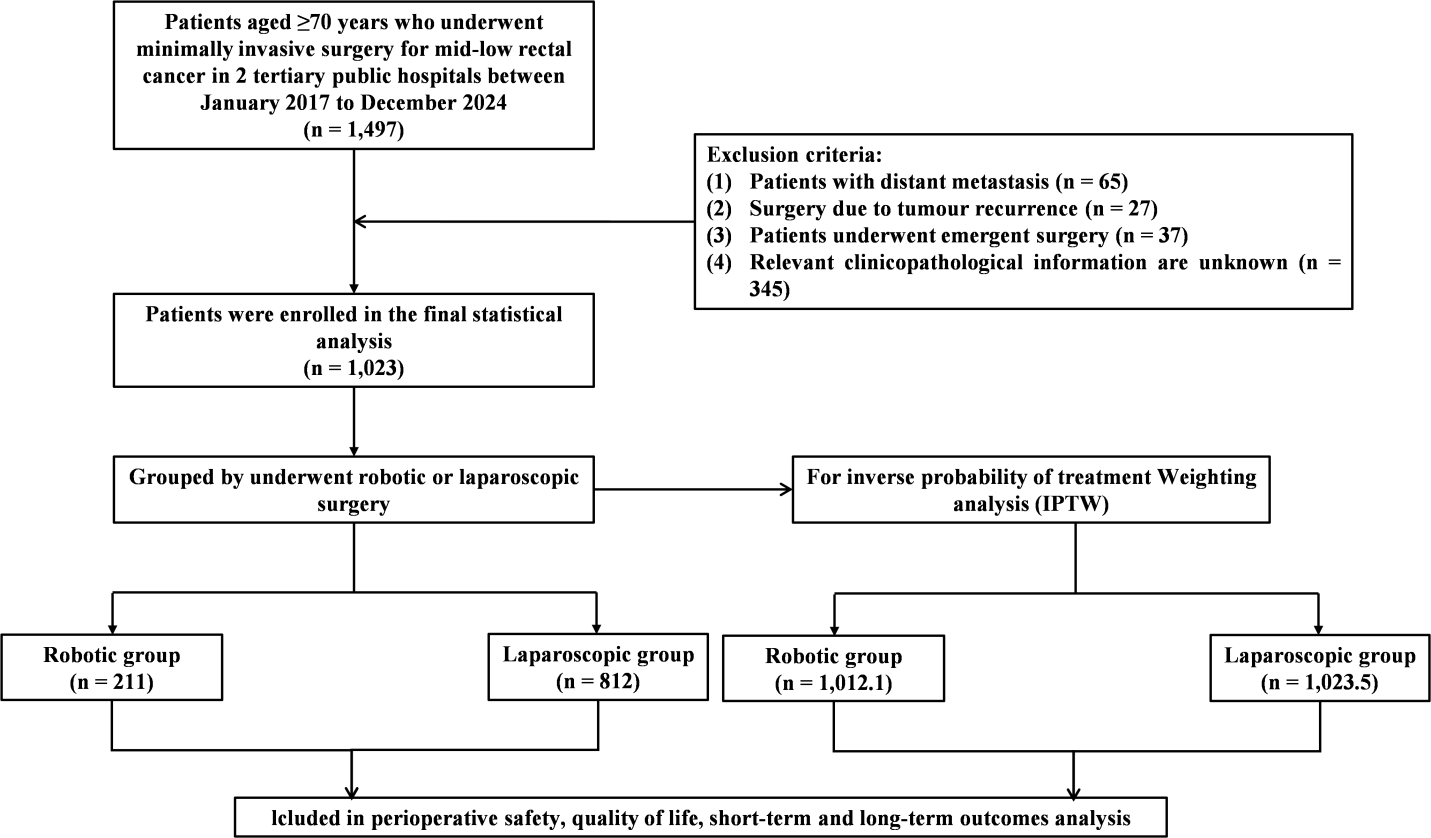
Flowchart of patient selection. IPTW, inverse probability of treatment weighting.

Eligible participants were selected by systematically reviewing the prospectively maintained colorectal cancer registry at our institution. The study preliminary included 1,497 RC patients who received minimally invasive resection between January 2017 and December 2024 at either the First Affiliated Hospital of Xi’an Jiaotong University or its affiliated Yulin Hospital. The inclusion criteria comprised: (1) age ≥70 years; (2) patients underwent robotic or laparoscopic surgery; (3) diagnosed with rectal adenocarcinoma through pathological examination; (4) tumor located ≤10 cm from the anal verge. Exclusion criteria consisted of: (1) presence of distant metastases; (2) surgery due to tumor recurrence; (3) underwent emergent surgery; (4) missing clinicopathological or survival data. After filtering, 1,023 patients were ultimately included in the study, with 211 patients in the robotic group (RG) and 812 patients in the laparoscopic group (LG). The overall flowchart is shown in **Figure 1**. The study protocol received approval from the ethics review boards of both participating institutions (2019-ZD-04).

### Surgical procedures

Among the two institutions, all participating surgeons had each performed over 300 laparoscopic RC resections and were formally trained and certified in robotic surgical techniques. All operations were performed in accordance with the expert consensus on robotic colorectal cancer surgery and adhered to the principles of TME (15). The selection of surgical approach depended on tumor base location, depth of invasion, as well as the preferences of the patients and their families.

The robotic and laparoscopic surgical approaches utilized five trocar placements. Key procedural phases included: (1) ligation of the inferior mesenteric artery at the appropriate location with concomitant lymph node dissection, (2) stepwise mobilization of the sigmoid and rectal segments with optional splenic flexure release, followed by lateral lymph node dissection when necessary, (3) distal rectal transection. Specimen retrieval was accomplished via abdominal, transanal, or transvaginal routes post complete resection. Reconstruction involved either end-to-end anastomosis or diversion procedures based on operative indications. All cases concluded with retro-anastomotic pelvic drainage placement.

### Assessment of quality of life

Each patient’s core QoL was assessed using the European Organization for Research and Treatment of Cancer Quality of Life Questionnaire for Colorectal Cancer 29 (16). The standardized Chinese version contains 4 functional domains (body image, anxiety, and male and female sexual function) and 9 symptom domains (urinary function, abdominal pain, stool characteristics, bowel function, stoma-related problems, bloating, dry mouth, hair loss, and taste alteration). Participants were asked to document any symptoms occurring within the previous weeks. All domain scores were converted to a 0–100 scale using linear transformation, with elevated values indicating worse functional status or increased symptom severity. QoL was assessed at three time points: before surgery, and at 6 and 12 months after surgery. Preoperative assessments were completed by patients themselves, while postoperative assessments were conducted via outpatient consultation or telephone follow-up.

### Oncological outcomes

The follow-up strategy was identical for both patient cohorts. Clinical assessments were planned every three months during the initial two years, semi-annually up to five years, and annually thereafter. The surveillance protocol included physical evaluation, laboratory tests, and radiographic studies. Follow-up data were censored in June 2025, with a median observation period of 40.0 months. Overall survival (OS) was defined as the time from surgical intervention until either mortality or the last follow-up. Cancer-specific survival (CSS) referred to the duration from resection to mortality directly related to RC.

### Inverse probability of treatment weighting (IPTW) analysis

To mitigate selection bias arising from the nonrandomized study design, a stabilized IPTW approach, utilizing propensity scores derived from covariate balancing, was applied to generate a balanced analytical cohort. Propensity scores for individual patients were derived through a binary logistic regression model to compute IPTW. And, the stabilized weights were applied to mitigate extreme weight values, minimize variance in the IPTW model, and control the standard errors of treatment effect estimates. In this study, baseline characteristics that may confound the relationship between exposure and outcome were included in the weight calculation, including age, sex, body mass index (BMI), tumor location, history of abdominal surgery, comorbidity, type of surgery, lateral lymph node dissection, American Society of Anesthesiologists score, clinical TNM stage, neoadjuvant therapy, and adjuvant therapy.

### Statistical analysis

Statistical analyses were performed using R 4.4.2 software. Categorical variables were compared using the *χ*^2^ test or Fisher’s exact test. Continuous variables were compared using Student’s *t* test or Mann–Whitney *U* test, depending on whether they were normally distributed. The Kaplan–Meier survival curves were used to compare the prognosis difference. A full-variable Cox regression model was used to explore prognostic factors affecting OS and CSS. IPTW was used to control for confounding in all analyses. Standardized mean differences (SMD) were used to evaluate balance before and after IPTW, where a standardized difference >10% was considered significant. P values were two-sided and significant when less than 0.05.

## Results

### Baseline characteristics of patients

1,023 eligible patients were eventually included in the study, comprising 211 who underwent robotic surgery and 812 treated laparoscopically. Following IPTW, the adjusted cohorts consisted of 1,012.1 and 1,023.5 patients in the two comparison groups, respectively.

Patient baseline characteristics pre- and post-IPTW are presented in **Table 1**. Prior to matching, significant disparities were identified between cohorts regarding age [74.3 ± 3.7 years vs. 75.6 ± 3.7 years; *P<*0.001], tumor location [5.4 ± 1.5 cm vs. 5.7 ± 1.6 cm; *P* = 0.045], and the proportion of neoadjuvant therapy [30.3% vs. 21.7%; *P* = 0.011] and adjuvant therapy [40.8% vs. 31.5%; *P* = 0.014]. Following IPTW adjustment, all baseline covariates achieved balance between groups (all *P*>0.05). This was further corroborated by SMD analysis, wherein post-matching SMD values were all below the 0.1 threshold, demonstrating effective covariate balance through IPTW (**Supplementary Figure 1**).

**Table 1:** Baseline characteristics of enrolled patients before and after IPTW.

Variable	Before IPTW		SMD	*p*-value	After IPTW		SMD	*p*-value
	RG (n = 211)	LG (n = 812)			RG (n = 1012.1)	LG(n = 1023.5)		
Age, years, mean±SD	74.3±3.7	75.6±3.7	0.350	<0.001	75.2±3.7	75.3±3.7	0.037	0.657
Sex, n (%)			0.032	0.732			0.016	0.850
Male	92 (43.6)	341 (42.0)			436.6 (43.1)	433.5 (42.4)		
Female	119 (56.4)	471 (58.0)			575.6 (56.9)	590.0 (57.6)		
ASA score, n (%)			0.113	0.172			0.018	0.836
I、II	136 (64.5)	479 (59.0)			598.0 (59.1)	613.5 (59.9)		
III、IV	75 (35.5)	333 (41.0)			414.2 (40.9)	410.0 (40.1)		
BMI, kg/m^2^, n (%)			0.097	0.250			0.013	0.882
≤25	158 (74.9)	573 (70.6)			717.0 (70.8)	730.9 (71.4)		
>25	53 (25.1)	239 (29.4)			295.2 (29.2)	292.6 (28.6)		
Distance to the anal verge, cm, mean±SD	5.4±1.5	5.7±1.6	0.156	0.045	5.7±1.6	5.7±1.6	0.007	0.932
Previous abdominal surgery, n (%)	27 (12.8)	83 (10.2)	0.081	0.342	104.3 (10.3)	109.5 (10.7)	0.013	0.866
Age-adjusted comorbidity index ≤ 2, n (%)	97 (46.0)	327 (40.3)	0.115	0.156	430.4 (42.5)	425.1 (41.5)	0.020	0.809
Type of surgery, n (%)			0.163	0.124			0.048	0.864
LAR	168 (79.6)	591 (72.8)			750.9 (74.2)	759.3 (74.2)		
ISR	32 (15.2)	159 (19.6)			200.1 (19.8)	191.5 (18.7)		
APR	11 (5.2)	62 (7.6)			61.1 (6.0)	72.7 (7.1)		
Lateral lymph node dissection, n (%)	17 (8.1)	38 (4.7)	0.077	0.139	56.7 (5.6)	55.8 (5.4)	0.007	0.926
Clinical TNM stage, n (%)			0.043	0.856			0.035	0.916
I	62 (29.4)	249 (30.7)			292.3 (28.9)	310.0 (30.3)		
II	85 (40.3)	332 (40.9)			414.7 (41.0)	417.8 (40.8)		
III	64 (30.3)	231 (28.4)			305.2 (30.1)	295.7 (28.9)		
Neoadjuvant therapy, n (%)	64 (30.3)	176 (21.7)	0.198	0.011	242.8 (24.0)	240.4 (23.5)	0.012	0.880
Adjuvant therapy, n (%)	86 (40.8)	256 (31.5)	0.193	0.014	354.4 (35.0)	342.5 (33.5)	0.033	0.685

IPTW, inverse probability of treatment weighting; RG, robotic group; LG, laparoscopic group; SMD, standardized mean differences; ASA, American society of Anesthesiologists; BMI, body mass index; LAR, low anterior resection; ISR, intersphincteric resection; APR, abdominoperineal resection; TNM, Tumor Node Metastasis.

### Comparison of postoperative short-term outcomes

Before matching, the median operation duration was significantly greater in the RG compared to the LG [220 min vs. 195 min; *P<*0.001]. When the docking and undocking time of the robotic system were excluded, the actual operation times for the two groups were comparable [201 min vs. 195 min; *P* = 0.147]. After IPTW adjustment, there was still no significant difference in the actual operation time [195 min vs. 191 min; *P* = 0.392]. However, after applying IPTW, the RG group demonstrated a significantly lower estimated blood loss [70 ml vs. 100 ml; *P<*0.001] and a reduced rate of intraoperative blood transfusion [7.2% vs. 12.7%; *P* = 0.031]. Regarding the comparison of postoperative recovery, pre-matching analysis revealed comparable postoperative recovery profiles between RG and LG, with the sole exception of significantly earlier urinary catheter removal in the RG [2 days vs. 3 days; *P* = 0.024]. Post-matching evaluation maintained this differential in catheter removal timing, while additionally demonstrating a statistically significant reduction in postoperative hospitalization duration for robotic-assisted cases [11 days vs. 13 days; *P* = 0.042] (**Table 2**).

**Table 2:** Comparison of surgical outcomes and postoperative recovery between robotic and laparoscopic surgery in elderly patients with mid-low rectal cancer before and after IPTW adjustment.

Variable	Before IPTW		*p*-value	After IPTW		*p*-value
	RG (n = 211)	LG (n = 812)		RG (n = 1012.1)	LG (n = 1023.5)	
Total operative time (min), median (IQR)	220 (180-255)	195 (150-240)	<0.001	215 (175-248)	191 (147-230)	<0.001
Docking time (min)	12 (8-15)	–	–	11 (8-14.5)	–	–
Undocking time (min)	6 (4-7)	–	–	6 (4-7)	–	–
Purely operating time (min)	201 (170-231)	195 (150-240)	0.147	195 (165-225)	191 (147-230)	0.392
Estimated blood loss (mL), median (IQR)	70 (50-100)	110 (50-130)	<0.001	70 (50-100)	100 (50-120)	<0.001
Intraoperative blood transfusion, n (%)	16 (7.6)	99 (12.2)	0.077	72.8 (7.2)	130.3 (12.7)	0.031
Construction of diverting ileostomy, n (%)	133 (63.0)	525 (64.7)	0.721	382.5 (37.8)	365.8 (35.7)	0.606
Days to first flatus (days), median (IQR)	2 (1-3)	2 (1-3)	0.472	2 (1-3)	2 (1-3)	0.541
Days to soft diet (days), median (IQR)	3 (2-4)	3.5 (2-5)	0.247	3 (2-4)	3 (2-5)	0.174
Postoperative hospital stay (days), median (IQR)	11(8-13)	12 (9-14)	0.117	11(8-13)	13 (9-14)	0.042
Postoperative urinary catheter removal time^*^ (days), median (IQR)	2 (1-4)	3 (1-4)	0.024	2 (1-4)	3 (1-4)	0.008

IPTW, inverse probability of treatment weighting; RG, robotic group; LG, laparoscopic group; IQR, inter-quartile range; *, For multiple catheterizations, the time of the last removal was recorded as the final time point.

Pathologic outcomes for both groups are summarized in **Table 3**. Before and after IPTW, no significant differences were identified in tumor size, proximal or distal resection margin distances, differentiation grade, pathological T or N stage, total lymph node yield, number of positive lymph nodes, quality of TME, or circumferential resection margin involvement (all *P* > 0.05).

**Table 3:** Comparison of pathologic outcomes between robotic and laparoscopic surgery in elderly patients with mid-low rectal cancer before and after IPTW.

Variable	Before IPTW		*p*-value	After IPTW		*p*-value
	RG (n = 211)	LG (n = 812)		RG (n = 1012.1)	LG (n = 1023.5)	
The size of tumor, (cm), mean±SD	3.6±1.2	3.7±1.3	0.559	3.6±1.3	3.7±1.3	0.444
Proximal margin, (cm), mean±SD	12.5±5.5	12.1±5.2	0.303	12.5±5.7	12.1±5.2	0.336
Distal margin, (cm), mean±SD	2.6±1.4	2.6±1.5	0.668	2.6±1.4	2.6±1.5	0.885
Grade of differentiation,no.(%)			0.112			0.097
Well	8 (3.8)	64 (7.9)		36.4 (3.6)	80.4 (7.9)	
Moderate	159 (75.3)	578 (71.2)		757.2 (74.8)	727.4 (71.1)	
Poor	44 (20.9)	170 (20.9)		218.5 (21.6)	215.8 (21.1)	
Pathological T stage, no.(%)			0.388			0.356
T1	33 (15.6)	111 (13.7)		152.2 (15.0)	137.8 (13.5)	
T2	34 (16.1)	168 (20.7)		161.6 (16.0)	209.8 (20.5)	
T3	120 (56.9)	429 (52.8)		590.2 (58.3)	543.5 (53.1)	
T4	24 (11.4)	104 (12.8)		108.1 (10.7)	132.5 (12.9)	
Pathological N stage, no.(%)			0.126			0.456
N0	128 (60.7)	541 (66.6)		627.5 (62.0)	672.5 (65.7)	
N1	48 (22.7)	176 (21.7)		226.6 (22.4)	225.6 (22.0)	
N2	35 (16.6)	95 (11.7)		158.1 (15.6)	125.4 (12.3)	
Retrieved LN, median ((IQR)	17 (13-19)	15 (12-18)	0.149	17 (13-19)	16 (12.5-18.8)	0.257
Positive LN, median ((IQR)	4 (1-6)	4 (1-5)	0.337	4 (1-6)	4 (1-6)	0.493
Quality of TME			0.147			0.284
Complete	199 (94.3)	741 (91.3)		956.7 (94.5)	944.4 (92.3)	
Nearly complete	12 (5.7)	71 (8.7)		55.5 (5.5)	79.1 (7.7)	
Positive circumferential resection no.(%)	8 (3.8)	36 (4.4)	0.827	39.6 (3.9)	43.5 (4.3)	0.835

IPTW, inverse probability of treatment weighting; RG, robotic group; LG, laparoscopic group; LN, lymph node; TME, total mesorectal excision.

Intraoperative and postoperative complications in both groups are described in **Table 4**. Before weighing, there were no statistically significant differences between RG and LG in terms of overall intraoperative complications and various types of intraoperative complications (all *P* > 0.05). After IPTW adjustment, the RG exhibited significantly reduced overall intraoperative complication rates [3.7% vs. 7.7%; *P* = 0.045]. Regarding the comparison of postoperative complications, following weight matching, the robotic cohort maintained significantly lower rates of both overall postoperative complications [32.4% vs. 40.7%; *P* = 0.043] and specific urinary retention events [2.1% vs. 6.0%; *P* = 0.021] compared to the laparoscopic cohort.

**Table 4:** Comparison of intraoperative and postoperative morbidity and mortality between robotic and laparoscopic surgery in elderly patients with mid-low rectal cancer before and after IPTW.

Variable	Before IPTW		*p*-value	After IPTW		*p*-value
	RG (n = 211)	LG (n = 812)		RG (n = 1012.1)	LG (n = 1023.5)	
Intraoperative complications, no. (%)	9 (4.3)	64 (7.9)	0.085	37.4 (3.7)	79.2 (7.7)	0.045
Mesenteric vascular injury	2 (0.9)	10 (1.2)	1.000	12.4 (1.2)	14.4 (1.4)	0.858
Presacral vascular injury	0 (0.0)	5 (0.6)	0.556	0 (0.0)	8.1 (0.8)	0.147
Bowel injury	3 (1.4)	12 (1.5)	1.000	26.0 (2.6)	15.6 (1.5)	0.421
Ureter injury	1 (0.5)	11 (1.4)	0.484	6.9 (0.7)	14.3 (1.4)	0.478
Vaginal perforation	1 (0.5)	9 (1.1)	0.659	4.9 (0.5)	11.4 (1.1)	0.419
Pelvic plexus nerve injury	0 (0.0)	7 (0.9)	0.376	0.0 (0.0)	12.4 (1.2)	0.138
Subcutaneous emphysema	2 (0.9)	10 (1.2)	1.000	15.2 (1.5)	13.1 (1.3)	0.836
Postoperative overall complications, no.(%)	64 (30.3)	331 (40.8)	0.007	328.2 (32.4)	416.7 (40.7)	0.043
Clavien-Dindo grade ≥ III	6 (2.8)	39 (4.8)	0.295	27.2 (2.7)	48.4 (4.7)	0.215
Anastomotic leakage	6 (2.8)	28 (3.4)	0.825	25.7 (2.5)	33.1 (3.2)	0.592
Intra-abdominal hemorrhage	6 (2.8)	34 (4.2)	0.485	30.6 (3.0)	40.8 (4.0)	0.554
Intestinal obstruction	9 (4.3)	31 (3.8)	0.921	45.6 (4.5)	36.8 (3.6)	0.564
Urinary infection	9 (4.3)	40 (4.9)	0.826	45.6 (4.5)	48.3 (4.7)	0.902
Urinary retention	5 (2.4)	51 (6.3)	0.040	21.4 (2.1)	61.1 (6.0)	0.021
Pelvic infection	3 (1.4)	15 (1.8)	0.901	13.1 (1.3)	18.0 (1.8)	0.636
Wound infection	12 (5.7)	56 (6.9)	0.636	61.8 (6.1)	67.9 (6.6)	0.799
Pulmonary infection	7 (3.3)	39 (4.8)	0.459	35.5 (3.5)	46.8 (4.6)	0.536
Cerebrovascular-related complications	5 (2.4)	29 (3.6)	0.514	20.7 (2.0)	34.8 (3.4)	0.294
Stoma-related complications	2 (0.9)	8 (1.0)	1.000	9.9 (1.0)	10.0 (1.0)	0.998
30-Day mortality	1 (0.5)	3 (0.4)	1.000	5.9 (0.6)	4.0 (0.4)	0.731

IPTW, inverse probability of treatment weighting; RG, robotic group; LG, laparoscopic group.

### Longitudinal assessment of changes in QoL

**Figure 2:**
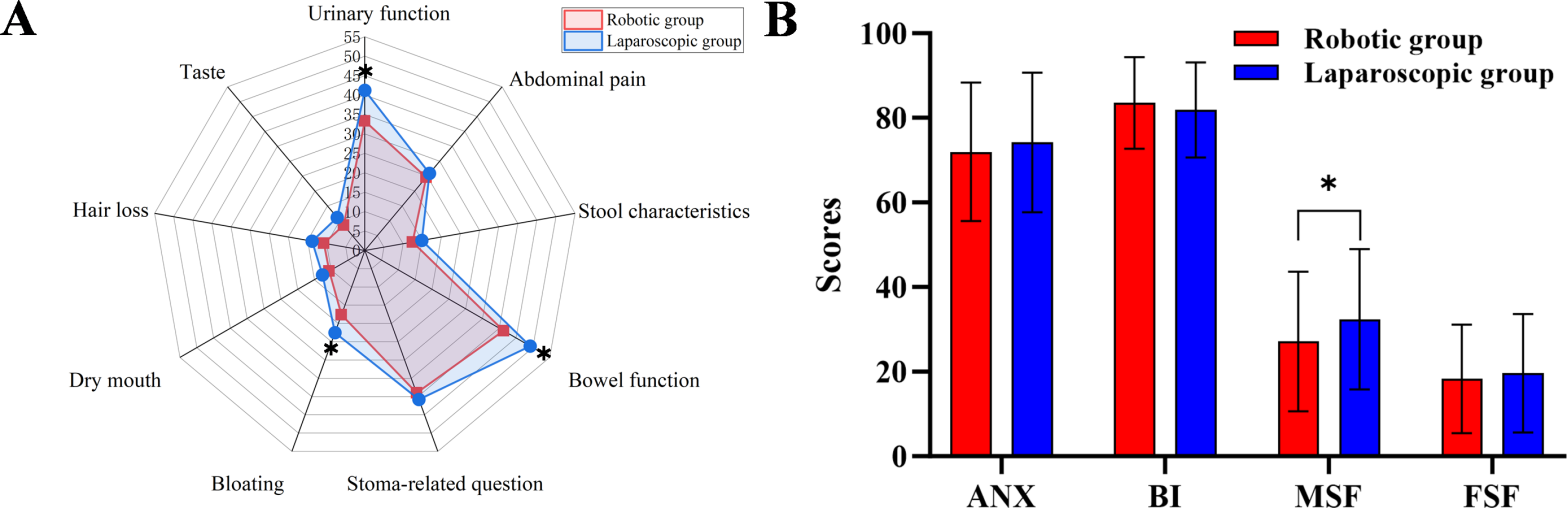
Comparative analysis of quality-of-life outcomes between robotic-assisted and laparoscopic surgery cohorts among older adults with mid-low rectal cancer at 6-month postoperative follow-up: **(A)** (symptom dimension), **(B)** (function dimension). ANX: anxiety; BI: body image; MSF: male sexual function; FSF: female sexual function; *: indicates significant difference with p < 0.05.

A total of 421 patients completed QoL assessments before surgery, at 6 months, and at 12 months postoperatively, including 132 in the RG and 289 in the LG. No significant differences were observed in any preoperative QoL domains between the two groups (**Supplementary Table 1**). The comparison of functional and symptomatic aspects of QoL 6 months after the surgery is shown in **Figure 2**. The symptom radar diagram shows that statistically significant intergroup differences in the QoL of patients 6 months after the surgery are manifested in urination [33.3 ± 13.2 vs. 41.2 ± 16.3; *P* < 0.001], bloating [17.6 ± 21.7 vs. 22.5 ± 20.7; *P* = 0.027] and defecation [41.3 ± 11.8 vs. 49.2 ± 12.9; *P* < 0.001] (**Figure 2A**). Furthermore, functional outcomes analysis indicated that male patients in the RG exhibited significantly better sexual function [27.1 ± 16.5 vs. 32.4 ± 16.6; *P* = 0.003] compared to those in the LG (**Figure 2B**). These differences persisted even 12 months postoperative assessment (**Supplementary Figure 2**). The detailed comparison of the postoperative QoL between the two groups is provided in **Supplementary Tables 2**, **3**.

### Comparison of long-term outcomes

**Figure 3:**
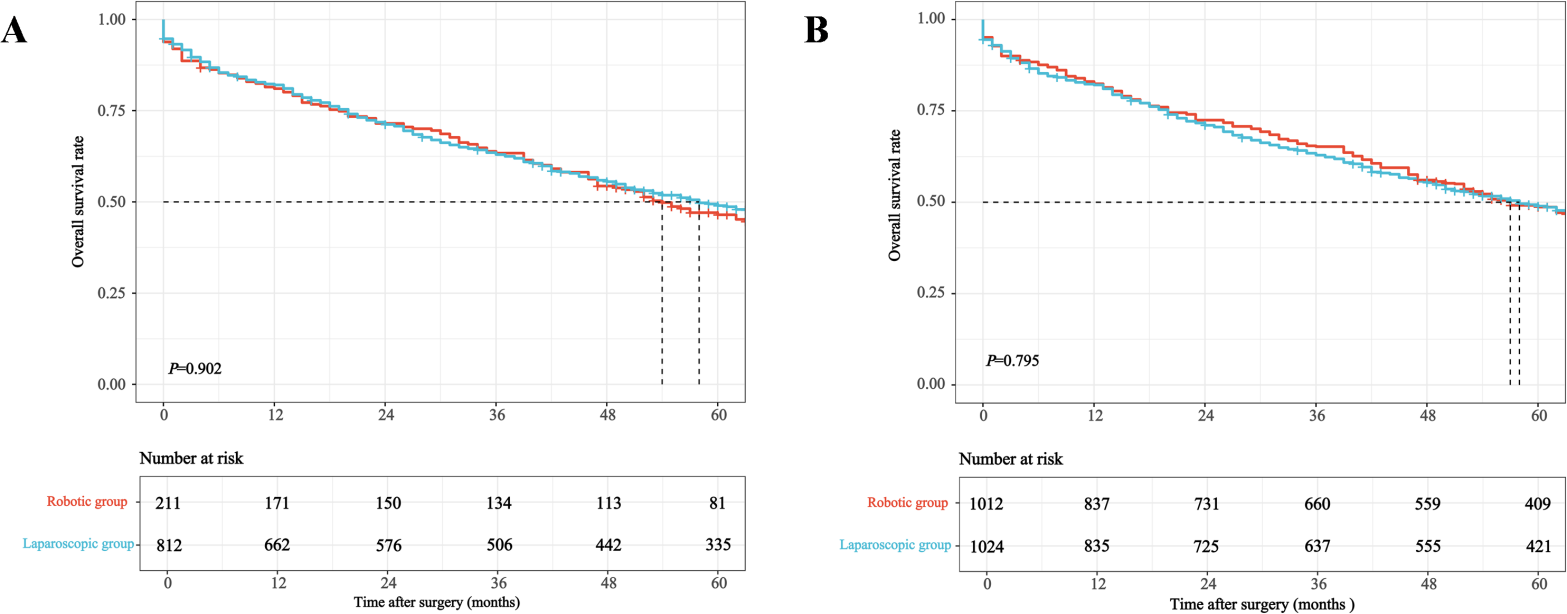
Kaplan-Meier curves for overall survival comparing robotic versus laparoscopic resection in older adults with mid-low rectal cancer before **(A)** and after **(B)** inverse probability of treatment weighting.

**Figure 4:**
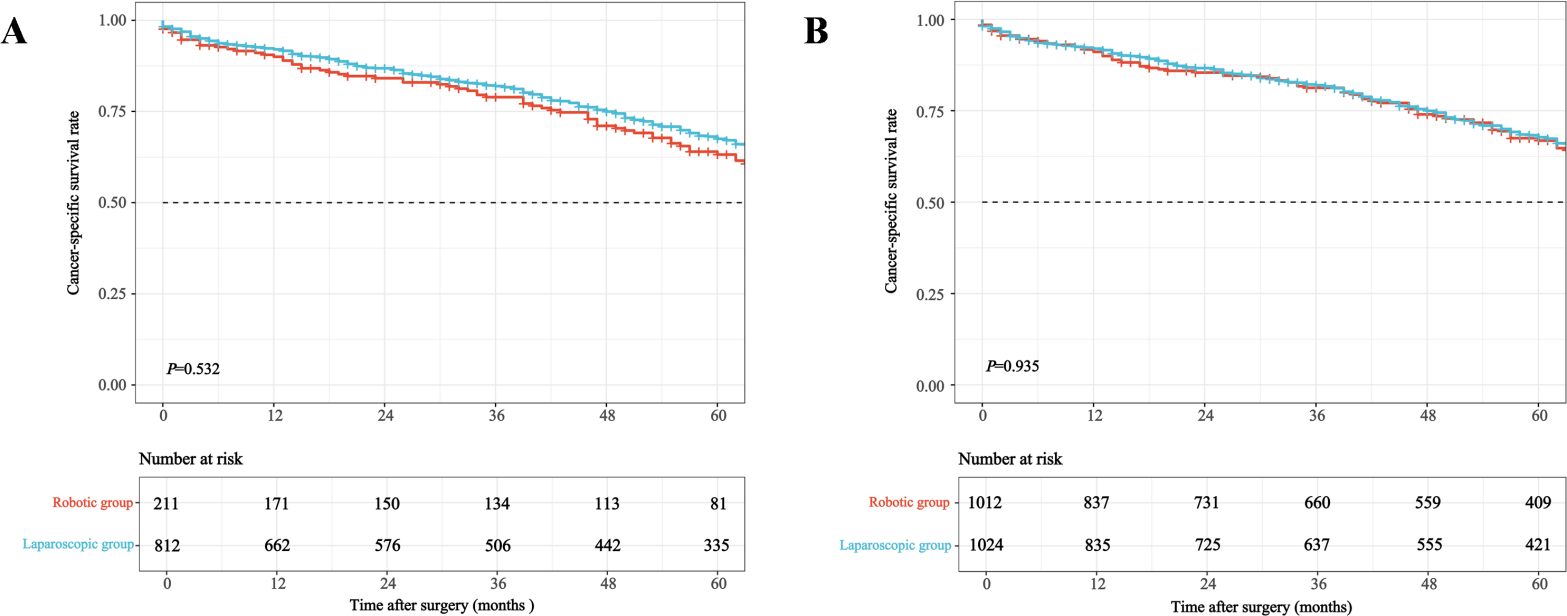
Kaplan-Meier curves for cancer-specific survival comparing robotic versus laparoscopic resection in older adults with mid-low rectal cancer before **(A)** and after **(B)** inverse probability of treatment weighting.

**Figure 5:**
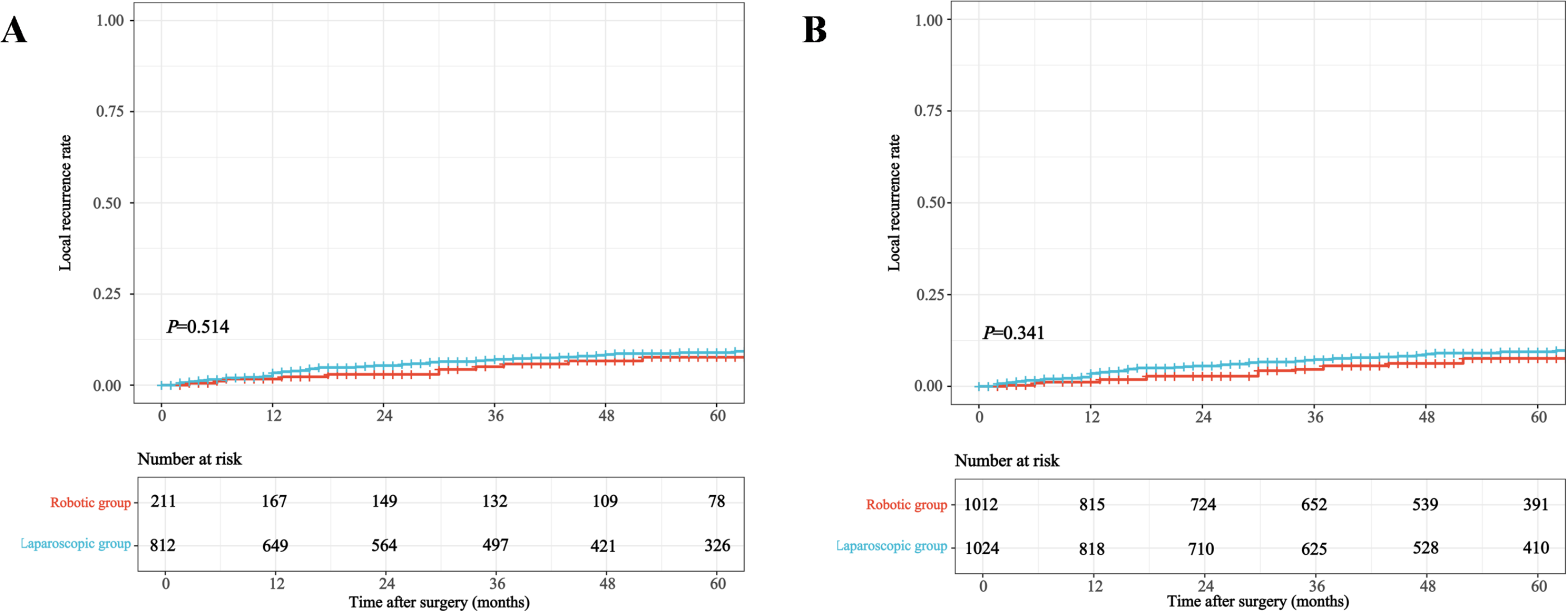
Kaplan-Meier curves for local recurrence comparing robotic versus laparoscopic resection in older adults with mid-low rectal cancer before **(A)** and after **(B)** inverse probability of treatment weighting.

Kaplan-Meier survival curves illustrating OS, CSS, and local recurrence before and after weighting are presented in **Figures 3**–**5**. After IPTW adjustment, no significant differences were found between RG and LG in 5-year OS (49.6% vs. 48.5%), CSS (64.7% vs. 64.3%), or local recurrence rates (7.2% vs. 8.9%) (all *P* > 0.05).

**Figure 6:**
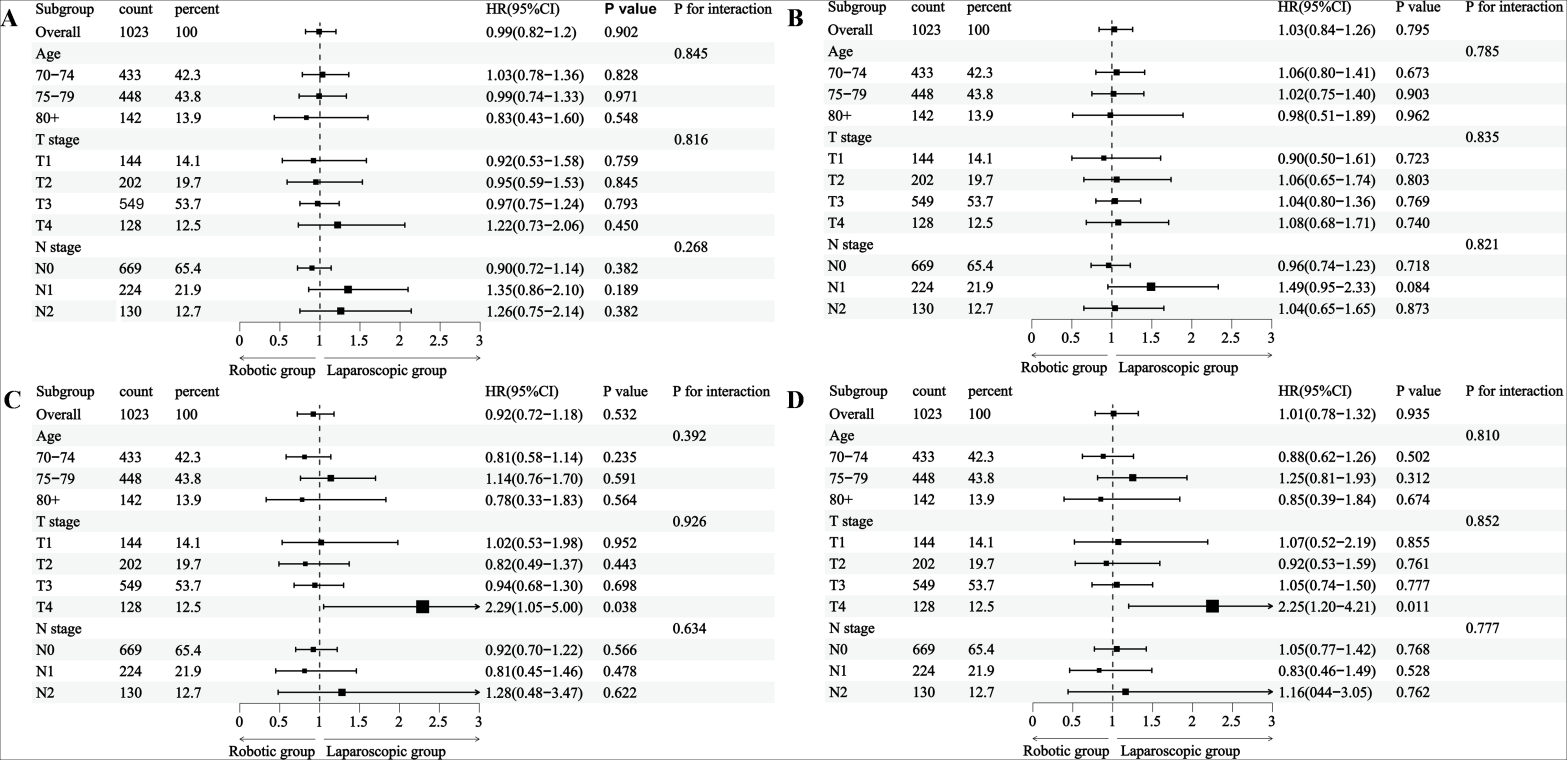
Interactions and subgroup analysis of OS and CSS: **(A)** OS before IPTW, **(B)** OS after IPTW, **(C)** CSS before IPTW, **(D)** CSS after IPTW. IPTW: inverse probability of treatment weighting.

After IPTW, no significant differences in OS [hazard ratio (HR)=1.03, 95%CI: 0.84-1.26, *P* = 0.795) and CSS (HR = 1.01, 95%CI: 0.78-1.32, *P* = 0.935) were observed between the groups (**Figure 6**). To further determine the effects of robotic vs. laparoscopic approach on various subgroups, we also conducted subgroup analyses according to different ages, pathological T stage, and N stage. Following propensity score weighting, no significant differences in OS or CSS were identified between the groups, either before or after adjustment, with the exception of a higher risk observed in the T4 stage subgroup for CSS (HR = 2.25, 95% CI: 1.20-4.21, *P* = 0.011) (**Figure 6**).

Multivariable regression models were further employed to assess the impact of variable interactions on the relations between surgical modality and patient prognosis. Whether confounding factors were unadjusted, adjusted for TNM stage alone, adjusted for significant factors in multivariate Cox analysis, or adjusted for other confounding factors, no statistically significant differences were observed in OS or CSS between the two groups (all *P* > 0.05) (**Supplementary Table 4**). Before and after IPTW, the results of univariate and multivariate analysis of OS and CSS are shown in **Supplementary Tables 5**–**8**.

## Discussion

Given the demographic shifts characterized by sustained population growth and accelerated societal aging, surgeons are increasingly confronted with the clinical challenge of managing geriatric patients diagnosed with mid-low RC. This epidemiological transition, coupled with their underrepresentation in clinical trials, underscores the imperative for developing optimized management strategies specifically designed for this vulnerable patient cohort. This multicenter retrospective study is the first to employ IPTW in comparing robotic-assisted and laparoscopic surgery in older adults with mid-low RC, specifically evaluating perioperative results, long-term survival, and QoL after surgery while minimizing confounding biases. Our findings demonstrate significant clinical advantages of the robotic approach, particularly in reducing intraoperative complications, preserving urogenital function, and potentially improving oncological outcomes in advanced T4-stage tumors. These results suggest that robotic surgery may offer a safer and functionally superior alternative for this vulnerable population.

Although the technical benefits of robotic-assisted TME over conventional laparoscopic resection for RC have been well documented since its adoption, the evidence remains particularly scarce regarding its application in elderly populations (17, 18). Notably, existing research on robotic rectal resection in this age demographic is limited both in quantity and scope. Most available studies primarily establish procedure safety through comparative analyses with younger cohorts (19–21), failing to provide robust evidence for its potential superiority to laparoscopic approaches in geriatric patients.

While previous meta-analyses consistently reported prolonged operative durations with robotic-assisted versus laparoscopic resection for RC (MH 27.32, *P* = 0.0004) (22), our IPTW-adjusted analysis revealed comparable pure operative times between approaches [195 min vs. 191 min; *P* = 0.392]. The observed total operation time disparity primarily stemmed from the installation and docking of the Da Vinci platform, along with the positioning and calibration of its robotic arms. Thus, the progressive accumulation of surgical experience and continuous refinement of robotic procedures are expected to lead to further optimization of total operative duration. Our study demonstrated statistically significant reductions in both the estimated intraoperative blood loss [70 ml vs. 100 ml; *P* < 0.001] and the incidence of intraoperative complications [3.7% vs. 7.7%; *P* = 0.045] in the RG compared to LG. This phenomenon can be largely attributed to the robotic system’s ability to mitigate the technical constraints associated with conventional laparoscopic instrumentation. Three-dimensional visualization together with enhanced dexterity of robotic arms have facilitated the identification and separation of mesorectal vessels and minimizes neurovascular bundle trauma. Moreover, the high-definition stereoscopic visualization of robotic surgery enabled consistent preservation of the mesorectal fascia and autonomic nerve plexuses, thereby reducing iatrogenic injury to adjacent organs. These clinical advantages are particularly evident in anatomically restricted situations, including male patients with excessively high BMI or those possessing a narrow pelvic structure.

Current evidence on oncologic results comparing robotic and laparoscopic techniques for RC is not yet definitive. To date, only one RCT (23) and limited retrospective series (24–26) have systematically compared the prognostic differences, with the majority demonstrating comparable survival outcomes. Notably, the groundbreaking REAL study recently revealed a statistically significant improvement in disease-free survival favoring robotic resection for middle or low RC (HR = 0.45, *P* = 0.03). These contradictory findings underscore the necessity for additional high-quality evidence to elucidate the potential long-term benefits of robotic-assisted approach in RC management. Furthermore, current literature lacks dedicated investigations comparing prognostic outcomes of these surgical modalities in elderly RC patients. Our IPTW-adjusted analysis revealed a statistically significant improvement in CSS with robotic approach exclusively in T4-stage geriatric cases. This selective benefit may be attributed to fundamental technical distinctions. Conventional laparoscopic instruments approach the pelvic cavity at near-vertical trajectories, creating restricted surgical fields and frequent instrument collision in narrow spaces - challenges exacerbated by deep-seated tumors or extensive mesorectal infiltration. In contrast, the robotic system’s upgraded visual system and hand tremor filtration allow precise dissection along embryological planes as well as achieving optimal mesorectal fascia preservation. In mid-low RC, the T4 stage is predominantly T4b, which is characterized by invasion into adjacent organs and complicates the achievement of a complete resection. Thus, these technical advantages culminate in higher-quality TME specimens which may explain the observed CSS benefit in advanced cases (27). Future validation of these results will require multicenter studies incorporating prolonged follow-up periods and standardized pathological evaluation criteria.

As cancer patient survival rates improve, the emphasis on QoL in determining treatment goals has increased. This study revealed that patients in the RG continued to exhibit significantly higher QoL scores one year postoperatively, with notable improvements particularly in urinary, defecatory, and male sexual function. These findings align with a recent meta-analysis conducted by Flynn et al., wherein robotic surgery was similarly associated with improved urinary function at the 12-month postoperative (28). In our prior cohort study, robotic-assisted surgery demonstrated sustained functional benefits for low RC patients, with significantly reduced incidence and severity of anorectal dysfunction through 18-month postoperative follow-up (29). In the LANDMARC study, the rate of erectile dysfunction after robotic rectal resection continued to be significantly reduced compared to the laparoscopic cohort (25.0% vs. 40.9%) at 1 year of follow-up (30). The findings of this study are consistent with previous research. However, in this study, no significant difference in postoperative sexual function was observed among female patients undergoing different surgical procedures. Several anatomical and technical factors may help explain this sex-based disparity. First, preservation of the hypogastric plexus relies heavily on the magnified view provided by minimally invasive platforms and meticulous intraoperative anatomical exposure. The broader female pelvis offers a relatively larger operative space for visualizing the pelvic autonomic nerves, thereby facilitating more accurate identification and preservation. Second, unlike in males, the anterior rectal wall in females contains no critical glandular structures such as the seminal vesicles or prostate; dissection of Denonvilliers’ fascia in males may involve these structures, contributing to a higher risk of postoperative sexual impairment. Third, in females, the pelvic autonomic nerves are typically situated within a triangular region bounded by the vagina, rectum, and levator ani muscle. These nerves run alongside vessels, forming neurovascular bundles that emerge from the posterolateral aspect of the vagina and extend laterally to innervate urogenital organs. As these structures gradually diverge from the rectal wall, the likelihood of direct intraoperative nerve injury is substantially lower in females than in males.

Several limitations should be acknowledged in this study. Firstly, although this represents the largest comparative study to date evaluating robotic versus laparoscopic surgery in older adults with mid-low RC, the sample size remains relatively modest. Secondly, the non-randomized retrospective design inherently carries risks of selection bias, despite our use of IPTW to mitigate confounding factors. Thirdly, the lack of a standardized definition for the “elderly” population across different regions and studies may limit the generalizability of our findings, warranting further validation in diverse geographic populations and specific age subgroups. Fourthly, the response rate to the questionnaires on QoL was relatively low. Consequently, future multicenter randomized controlled trials with larger cohorts focused specifically on elderly patients with mid-low RC are warranted to corroborate these findings.

## Conclusion

In summary, this IPTW-based study indicates that robotic-assisted surgery is a safe, feasible, and efficacious option for older patients with mid-low RC, offering superior perioperative outcomes relative to conventional laparoscopy. These advantages include reduced blood loss, lower rates of intra- and postoperative complications, and enhanced preservation of genitourinary function. While demonstrating comparable OS and CSS between the two groups, the robotic approach showed significantly improved CSS in T4 tumors, necessitating extended follow-up confirmation.

## Data Availability

The raw data supporting the conclusions of this article will be made available by the authors, without undue reservation.
