# Supplementary material for: Comparison of clinical outcomes and quality of life for robotic versus laparoscopic surgery in elderly patients with mid-low rectal cancer: a multicenter cohort study with inverse probability of treatment weighting analysis
Source: Front Oncol. 2026 Apr 21;16:1809161. doi: 10.3389/fonc.2026.1809161 (PMC13138923; doi:10.3389/fonc.2026.1809161)
Supplement: Supplementary file 1 [file SupplementaryFile1.docx]

**Comparison of clinical outcomes and quality of life for robotic versus laparoscopic surgery in elderly patients with mid-low rectal cancer: a multicenter cohort study with inverse probability of treatment weighting analysis**

Zhang et al.

(Supplementary materials)

**
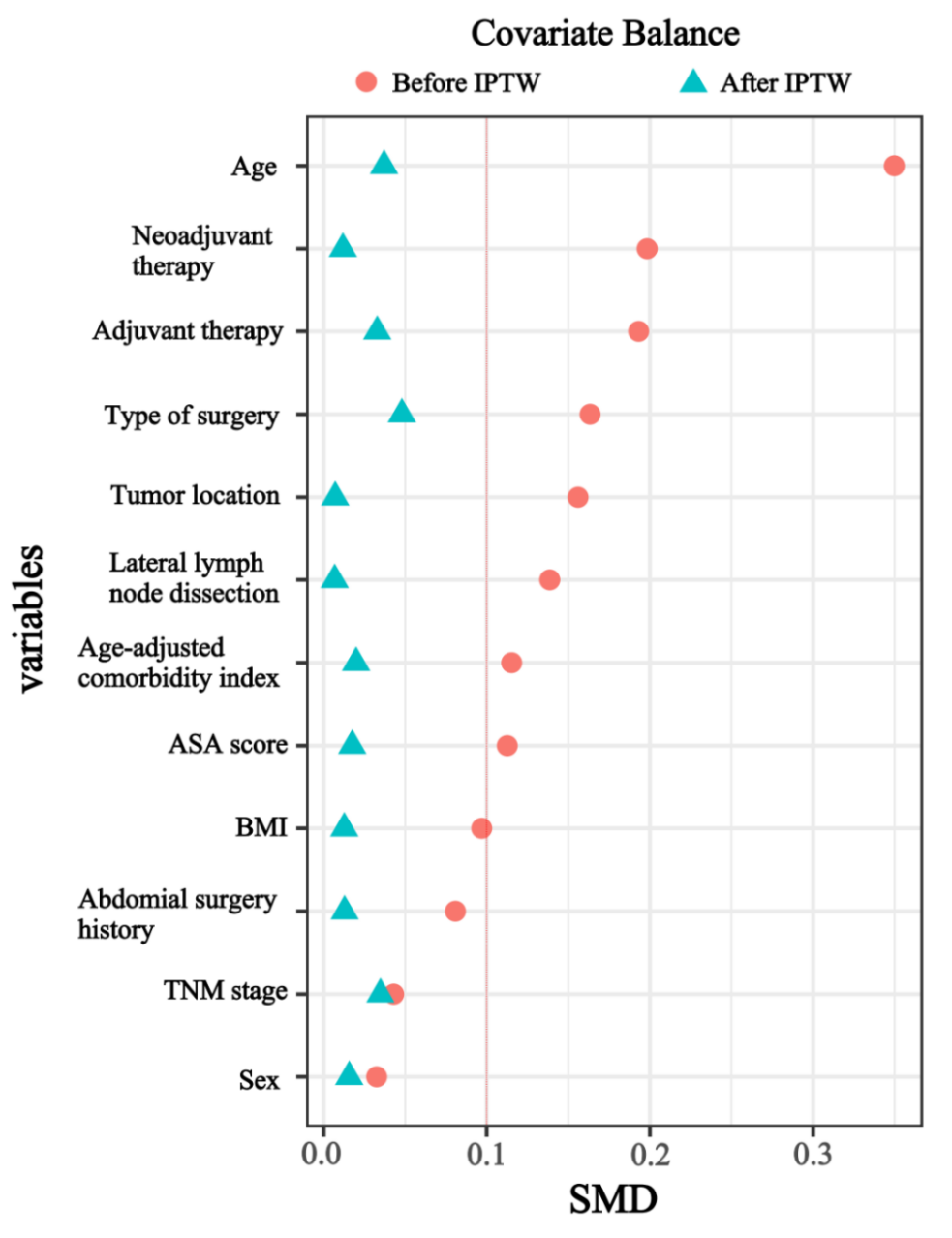
**

**Supplementary Figure. 1** Standardized mean differences for baseline information between the two groups before and after inverse probability of treatment weighting.

After weighting by IPTW, the SMD for all baseline variables were below 10%, showing that the variables were more comparable. IPTW: inverse probability

of treatment weighting, SMD: standardized mean difference


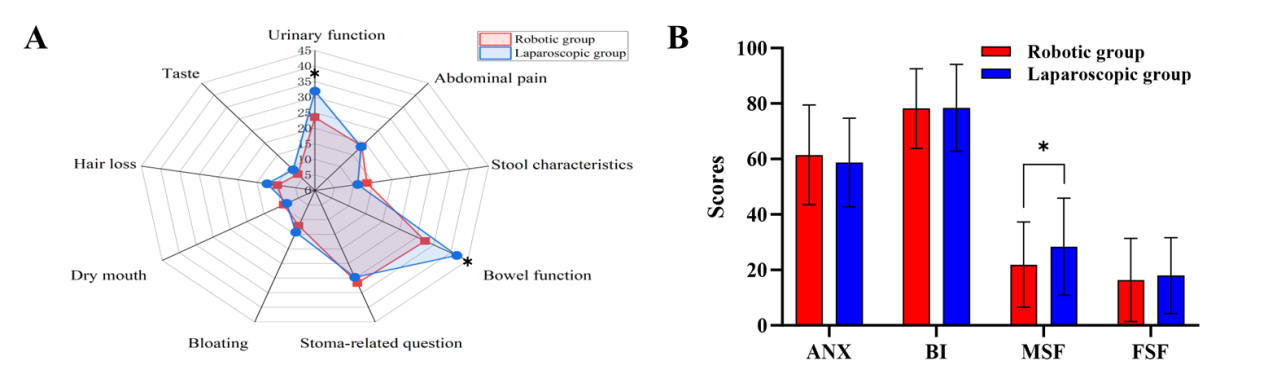


**Supplementary Figure. 2** Comparative analysis of quality-of-life outcomes between robotic-assisted and laparoscopic surgery groups in elderly patients with mid-low rectal cancer at 12-month postoperative follow-up: A (symptom dimension), B (function dimension). ANX: anxiety; BI: body image; MSF: male sexual function; FSF: female sexual function; ; *: indicates significant difference with p < 0.05.

**Supplementary Table 1** Quality of life of elderly patients with mid-low rectal cancer before surgery according to EORTC QLQ-CR29.

|  | | Robotic group  (n = 132) | Laparoscopic group  (n = 289) | *p*-value |
| --- | --- | --- | --- | --- |
| Functional evaluation | |  |  |  |
| Anxiety | | 65.1±11.9 | 65.9±13.1 | 0.547 |
|  | Body image | 77.8±12.6 | 76.0±11.5 | 0.167 |
|  | Male sexual function^a^ | 15.6±10.6 | 13.9±11.6 | 0.176 |
| Female sexual function^b^ | | 11.6±8.6 | 10.6±8.2 | 0.229 |
| Symptom evaluation | |  |  |  |
|  | Urinary function | 14.5±5.3 | 15.3±6.1 | 0.177 |
| Abdominal pain | | 4.9±2.3 | 5.2±2.4 | 0.237 |
|  | Stool characteristics | 3.9±2.5 | 4.3±2.1 | 0.150 |
|  | Bloating | 25.5±13.9 | 24.4±18.4 | 0.548 |
| Dry mouth | | 9.1±8.6 | 9.9±8.4 | 0.442 |
| Hair loss | | 10.8±14.1 | 11.8±14.2 | 0.494 |
| Taste | | 8.6±13.4 | 9.9±13.7 | 0.371 |
| Bowel function | | 15.1±8.3 | 14.2±8.8 | 0.326 |
|  | Stoma-related^c^ | - | - | - |
|  | ^a^:169 individuals completed the questionnaire;  ^b^:252 individuals completed the questionnaire;  ^c^:0 individuals completed the questionnaire. | | | |

**Supplementary Table 2** Quality of life of elderly patients with mid-low rectal cancer after 6 months of treatment according to EORTC QLQ-CR29.

|  | | Robotic group  (n = 132) | Laparoscopic group  (n = 289) | *p*-value |
| --- | --- | --- | --- | --- |
| Functional evaluation | |  |  |  |
| Anxiety | | 72.0±16.4 | 74.2±16.5 | 0.201 |
|  | Body image | 83.6±10.9 | 81.9±11.3 | 0.148 |
|  | Male sexual function^a^ | 27.1±16.5 | 32.4±16.6 | 0.003 |
| Female sexual function^b^ | | 18.3±12.8 | 19.6±14.0 | 0.335 |
| Symptom evaluation | |  |  |  |
|  | Urinary function | 33.3±13.2 | 41.2±16.3 | ＜0.001 |
| Abdominal pain | | 24.6±11.3 | 25.9±12.0 | 0.284 |
|  | Stool characteristics | 12.5±16.9 | 14.9±19.0 | 0.201 |
|  | Bloating | 17.6±21.7 | 22.5±20.7 | 0.027 |
| Dry mouth | | 10.6±16.0 | 12.6±16.8 | 0.239 |
| Hair loss | | 10.7±17.4 | 13.8±16.9 | 0.084 |
| Taste | | 8.5±16.8 | 11.1±17.4 | 0.162 |
| Bowel function | | 41.3±11.8 | 49.2±12.9 | ＜0.001 |
|  | Stoma-related^c^ | 39.0±12.4 | 40.8±13.0 | 0.169 |
|  | ^a^:169 individuals completed the questionnaire;  ^b^:252 individuals completed the questionnaire;  ^c^:93 individuals completed the questionnaire. | | | |

**Supplementary Table 3** Quality of life of elderly patients with mid-low rectal cancer after 12 months of treatment according to EORTC QLQ-CR29.

|  | | Robotic group  (n = 132) | Laparoscopic group  (n = 289) | *p*-value |
| --- | --- | --- | --- | --- |
| Functional evaluation | |  |  |  |
| Anxiety | | 61.4±18.0 | 58.7±16.0 | 0.127 |
|  | Body image | 78.2±14.4 | 78.4±15.7 | 0.868 |
|  | Male sexual function^a^ | 21.9±15.4 | 28.3±17.5 | 0.001 |
| Female sexual function^b^ | | 16.4±15.0 | 18.0±13.7 | 0.365 |
| Symptom evaluation | |  |  |  |
|  | Urinary function | 23.5±10.0 | 31.9±11.8 | ＜0.001 |
| Abdominal pain | | 18.6±9.8 | 18.3±9.5 | 0.791 |
|  | Stool characteristics | 13.5±23.0 | 11.1±19.7 | 0.286 |
|  | Bloating | 12.1±19.6 | 14.3±22.6 | 0.328 |
| Dry mouth | | 9.2±14.4 | 8.3±16.2 | 0.585 |
| Hair loss | | 9.7±17.1 | 12.4±18.5 | 0.157 |
| Taste | | 6.8±13.4 | 8.7±13.1 | 0.164 |
| Bowel function | | 32.5±10.9 | 41.8±14.4 | ＜0.001 |
|  | Stoma-related^c^ | 31.7±12.6 | 29.7±10.2 | 0.089 |
|  | ^a^:169 individuals completed the questionnaire;  ^b^:252 individuals completed the questionnaire;  ^c^:67 individuals completed the questionnaire. | | | |

**Supplementary Table 4** The relationship between surgical methods and overall survival as well as cancer-specific survival

| Variable | | | Approach |  |  |
| --- | --- | --- | --- | --- | --- |
|  |  |  | RG | LG | *p*-value |
| Before IPTW | OS | Crude HR (95% CI) | 1.00 (reference) | 0.99 (0.82-1.20) | 0.902 |
|  |  | HR (95% CI)* | 1.00 (reference) | 1.01 (0.84-1.22) | 0.886 |
|  |  | HR (95% CI)** | 1.00 (reference) | 1.04 (0.86-1.26) | 0.712 |
|  |  | HR (95% CI)*** | 1.00 (reference) | 1.03 (0.84-1.25) | 0.792 |
|  | CSS | Crude HR (95% CI) | 1.00 (reference) | 0.92 (0.72-1.18) | 0.532 |
|  |  | HR (95% CI)* | 1.00 (reference) | 0.93 (0.72-1.19) | 0.597 |
|  |  | HR (95% CI)** | 1.00 (reference) | 0.90 (0.70-1.15) | 0.414 |
|  |  | HR (95% CI)*** | 1.00 (reference) | 0.92 (0.71-1.18) | 0.522 |
| After IPTW | OS | Crude HR (95% CI) | 1.00 (reference) | 1.03 (0.84-1.26) | 0.795 |
|  |  | HR (95% CI)* | 1.00 (reference) | 1.04 (0.85-1.28) | 0.674 |
|  |  | HR (95% CI)** | 1.00 (reference) | 1.08 (0.89-1.34) | 0.497 |
|  |  | HR (95% CI)*** | 1.00 (reference) | 1.04 (0.85-1.27) | 0.730 |
|  | CSS | Crude HR (95% CI) | 1.00 (reference) | 1.01 (0.78-1.32) | 0.935 |
|  |  | HR (95% CI)* | 1.00 (reference) | 1.04 (0.81-1.37) | 0.613 |
|  |  | HR (95% CI)** | 1.00 (reference) | 1.07 (0.84-1.39) | 0.501 |
|  |  | HR (95% CI)*** | 1.00 (reference) | 1.00 (0.77-1.31) | 0.981 |
| IPTW, inverse probability of treatment weighting; RG, robotic group; LG, laparoscopic group; OS, overall survival; CSS, cancer-specific survival; HR, hazard ratio.  *Adjusted for pTNM;  **Adjusted for age, age-adjusted comorbidity index, pT, pN, differentiation, and adjuvant therapy;  ***Adjusted for age, sex, ASA score, BMI, age-adjusted comorbidity index, tumor location, history of abdominal surgery, type of surgery, lateral lymph node dissection, tumor size, differentiation, pT, pN, quality of total mesorectal excision, R0 resection, neoadjuvant therapy, adjuvant therapy. | | | | | |

**Supplementary Table 5** Univariate and multivariate analyses of risk factors for overall survival in elderly patients with mid-low rectal cancer before inverse probability of treatment weighting.

| **Variables** | | | **Univariate analysis** | |  | **Multivariate analysis** | |
| --- | --- | --- | --- | --- | --- | --- | --- |
|  |  |  | **HR (95% CI)** | ***p*-value** |  | **HR (95% CI)** | ***p*-value** |
| Age | | |  | ＜0.001 |  |  | 0.001 |
|  | 70-74 | | Reference |  |  | Reference |  |
|  | 75-79 | | 1.143 (0.957-1.365) |  |  | 1.156 (0.966-1.383) |  |
|  | 80+ | | 1.539 (1.248-1.897) |  |  | 1.514 (1.227-1.868) |  |
| Sex | | |  | 0.211 |  |  |  |
|  | male | | Reference |  |  |  |  |
|  | female | | 1.108 (0.943-1.302) |  |  |  |  |
| ASA score | | |  | 0.175 |  |  |  |
|  | I or II | | Reference |  |  |  |  |
|  | III or IV | | 1.114 (0.953-1.302) |  |  |  |  |
| Age-adjusted comorbidity index ≤ 2 | | |  | 0.001 |  |  |  |
|  | Yes | | Reference |  |  |  |  |
|  | No | | 1.572 (1.214-2.036) |  |  |  |  |
| BMI, kg/m^2^ | | |  | 0.506 |  |  |  |
|  | ≤25 | | Reference |  |  |  |  |
|  | ＞25 | | 0.944 (0.796-1.119) |  |  |  |  |
| History of abdominal surgery | | |  | 0.850 |  |  |  |
|  | No | | Reference |  |  |  |  |
|  | Yes | | 1.024 (0.801-1.308) |  |  |  |  |
| Lateral lymph node dissection | | |  | 0.201 |  |  |  |
|  | No | | Reference |  |  |  |  |
|  | Yes | | 1.140 (0.933-1.394) |  |  |  |  |
| Distance to the anal verge, cm | | |  | 0.509 |  |  |  |
|  | ＞5 | | Reference |  |  |  |  |
|  | ≤5 | | 0.906 (0.677-1.213) |  |  |  |  |
| Differentiation grade | | |  | 0.005 |  |  |  |
|  | Well | | Reference |  |  |  |  |
|  | Moderate | | 1.159 (0.822-1.635) |  |  |  |  |
|  | Poor | | 1.532 (1.062-2.210) |  |  |  |  |
| Type of surgery | | |  | 0.945 |  |  |  |
|  | LAR | | Reference |  |  |  |  |
|  | ISR | | 0.965 (0.789-1.180) |  |  |  |  |
|  | APR | | 1.027 (0.759-1.391) |  |  |  |  |
| Surgical approach | | |  | 0.902 |  |  |  |
|  | Robotic surgery | | Reference |  |  |  |  |
|  | Laparoscopic surgery | | 0.988 (0.817-1.196) |  |  |  |  |
| p T stage | | |  | ＜0.001 |  |  | ＜0.001 |
|  | T1 | | Reference |  |  | Reference |  |
|  | T2 | | 1.317 (0.987-1.765) |  |  | 1.305 (0.978-1.743) |  |
|  | T3 | | 1.535 (1.169-1.970) |  |  | 1.736 (1.039-2.902) |  |
|  | T4 | | 2.657 (1.969-3.585) |  |  | 2.676 (1.555-4.604) |  |
| p N stage | | |  | ＜0.001 |  |  | 0.006 |
|  | N0 | | Reference |  |  | Reference |  |
|  | N1 | | 1.336 (1.099-1.624) |  |  | 1.679 (0.890-3.159) |  |
|  | N2 | | 2.118 (1.669-2.687) |  |  | 2.319 (1.252-4.298) |  |
| Tumor size, mm | | |  | 0.141 |  |  |  |
|  | | ≤40 | Reference |  |  |  |  |
|  | | ＞40 | 0.870 (0.723-1.047) |  |  |  |  |
| Quality of TME | | |  | 0.076 |  |  |  |
|  | | Complete | Reference |  |  |  |  |
|  | | Nearly complete | 1.289 (0.974-1.708) |  |  |  |  |
| R0 resection | | |  | ＜0.001 |  |  | ＜0.001 |
|  | | Yes | Reference |  |  | Reference |  |
|  | | No | 3.466 (2.540-4.729) |  |  | 2.929(2.111-4.065) |  |
| Neoadjuvant therapy | | |  | 0.055 |  |  |  |
|  | | No | Reference |  |  |  |  |
|  | | Yes | 0.686 (0.497-1.009) |  |  |  |  |
| Adjuvant therapy | | |  | ＜0.001 |  |  | 0.001 |
|  | No | | Reference |  |  | Reference |  |
|  | Yes | | 0.760 (0.652-0.887) |  |  | 0.763 (0.653-0.892) |  |
| HR, hazard ratio; ASA, American society of Aneshesiologists; BMI, body mass index; LAR, low anterior resection; ISR, intersphincteric resection; APR, abdominoperineal resection; TME, total mesorectal excision. | | | | | | | |

**Supplementary Table 6** Univariate and multivariate analyses of risk factors for overall survival in elderly patients with mid-low rectal cancer after inverse probability of treatment weighting.

| **Variables** | | | **Univariate analysis** | |  | **Multivariate analysis** | |
| --- | --- | --- | --- | --- | --- | --- | --- |
|  |  |  | **HR (95% CI)** | ***p*-value** |  | **HR (95% CI)** | ***p*-value** |
| Age | | |  | ＜0.001 |  |  | ＜0.001 |
|  | 70-74 | | Reference |  |  | Reference |  |
|  | 75-79 | | 1.135 (0.904-1.425) |  |  | 1.172 (0.937-1.465) |  |
|  | 80+ | | 1.578 (1.202-2.072) |  |  | 1.552 (1.186-2.031) |  |
| Sex | | |  | 0.317 |  |  |  |
|  | male | | Reference |  |  |  |  |
|  | female | | 1.113 (0.903-1.373) |  |  |  |  |
| ASA score | | |  | 0.249 |  |  |  |
|  | I or II | | Reference |  |  |  |  |
|  | III or IV | | 1.128 (0.919-1.385) |  |  |  |  |
| Age-adjusted comorbidity index ≤ 2 | | |  | 0.021 |  |  |  |
|  | Yes | | Reference |  |  | Reference | ＜0.001 |
|  | No | | 1.297 (1.040-1.619) |  |  | 1.325 (1.059-1.658) |  |
| BMI, kg/m^2^ | | |  | 0.641 |  |  |  |
|  | ≤25 | | Reference |  |  |  |  |
|  | ＞25 | | 0.946 (0.748-1.196) |  |  |  |  |
| History of abdominal surgery | | |  | 0.513 |  |  |  |
|  | No | | Reference |  |  |  |  |
|  | Yes | | 1.114 (0.807-1.538) |  |  |  |  |
| Lateral lymph node dissection | | |  | 0. 213 |  |  |  |
|  | No | | Reference |  |  |  |  |
|  | Yes | | 1.240 (0.884-1.740) |  |  |  |  |
| Distance to the anal verge, cm | | |  | 0.333 |  |  |  |
|  | ＞5 | | Reference |  |  |  |  |
|  | ≤5 | | 0.818 (0.545-1.228) |  |  |  |  |
| Differentiation grade | | |  | 0.347 |  |  |  |
|  | Well | | Reference |  |  |  |  |
|  | Moderate | | 1.001 (0.632-1.612) |  |  |  |  |
|  | Poor | | 1.396 (0.844-2.309) |  |  |  |  |
| Type of surgery | | |  | 0.978 |  |  |  |
|  | LAR | | Reference |  |  |  |  |
|  | ISR | | 0.997 (0.755-1.316) |  |  |  |  |
|  | APR | | 1.056 (0.718-1.553) |  |  |  |  |
| Surgical approach | | |  | 0.795 |  |  |  |
|  | Robotic surgery | | Reference |  |  |  |  |
|  | Laparoscopic surgery | | 1.027 (0.839-1.258) |  |  |  |  |
| p T stage | | |  | ＜0.001 |  |  | ＜0.001 |
|  | T1 | | Reference |  |  | Reference |  |
|  | T2 | | 1.227 (0.846-1.779) |  |  | 1.216 (0.842-1.757) |  |
|  | T3 | | 1.457 (1.047-2.029) |  |  | 1.343 (0.965-1.870) |  |
|  | T4 | | 2.726 (1.813-4.099) |  |  | 2.254 (1.477-3.439) |  |
| p N stage | | |  | ＜0.001 |  |  | ＜0.001 |
|  | N0 | | Reference |  |  | Reference |  |
|  | N1 | | 1.200 (0.920-1.566) |  |  | 1.085 (0.829-1.421) |  |
|  | N2 | | 2.241 (1.663-3.020) |  |  | 1.892 (1.379-2.596) |  |
| Tumor size, mm | | |  | 0.022 |  |  |  |
|  | | ≤40 | Reference |  |  |  |  |
|  | | ＞40 | 1.269 (1.035-1.556) |  |  |  |  |
| Quality of TME | | |  | 0.097 |  |  |  |
|  | | Complete | Reference |  |  |  |  |
|  | | Nearly complete | 1.352 (0.947-1.929) |  |  |  |  |
| R0 resection | | |  | ＜0.001 |  |  | ＜0.001 |
|  | | Yes | Reference |  |  | Reference |  |
|  | | No | 3.262 (2.637-4.037) |  |  | 2.851 (2.198-3.698) |  |
| Neoadjuvant therapy | | |  | 0.242 |  |  |  |
|  | | No | Reference |  |  |  |  |
|  | | Yes | 0.774 (0.504-1.189) |  |  |  |  |
| Adjuvant therapy | | |  | 0.081 |  |  |  |
|  | No | | Reference |  |  |  |  |
|  | Yes | | 0.834 (0.680-1.022) |  |  |  |  |
| HR, hazard ratio; ASA, American society of Aneshesiologists; BMI, body mass index; LAR, low anterior resection; ISR, intersphincteric resection; APR, abdominoperineal resection; TME, total mesorectal excision. | | | | | | | |

**Supplementary Table 7** Univariate and multivariate analyses of risk factors for cancer-specific survival in elderly patients with mid-low rectal cancer before inverse probability of treatment weighting.

| **Variables** | | | **Univariate analysis** | |  | **Multivariate analysis** | |
| --- | --- | --- | --- | --- | --- | --- | --- |
|  |  |  | **HR (95% CI)** | ***p*-value** |  | **HR (95% CI)** | ***p*-value** |
| Age | | |  | 0.038 |  |  | 0.050 |
|  | 70-74 | | Reference |  |  | Reference |  |
|  | 75-79 | | 1.104 (0.838-1.452) |  |  | 1.190 (0.900-1.572) |  |
|  | 80+ | | 1.503 (1.090-2.072) |  |  | 1.495 (1.083-2.064) |  |
| Sex | | |  | 0.751 |  |  |  |
|  | male | | Reference |  |  |  |  |
|  | female | | 0.960 (0.744-1.238) |  |  |  |  |
| ASA score | | |  | 0.274 |  |  |  |
|  | I or II | | Reference |  |  |  |  |
|  | III or IV | | 1.144 (0.899-1.455) |  |  |  |  |
| Age-adjusted comorbidity index ≤ 2 | | |  | ＜0.001 |  |  |  |
|  | Yes | | Reference |  |  |  |  |
|  | No | | 2.172 (1.541-3.062) |  |  |  |  |
| BMI, kg/m^2^ | | |  | 0.451 |  |  |  |
|  | ≤25 | | Reference |  |  |  |  |
|  | ＞25 | | 1.103 (0.854-1.425) |  |  |  |  |
| History of abdominal surgery | | |  | 0.477 |  |  |  |
|  | No | | Reference |  |  |  |  |
|  | Yes | | 1.141 (0.793-1.642) |  |  |  |  |
| Lateral lymph node dissection | | |  | 0.754 |  |  |  |
|  | No | | Reference |  |  |  |  |
|  | Yes | | 1.052 (0.766-1.445) |  |  |  |  |
| Distance to the anal verge, cm | | |  | 0.723 |  |  |  |
|  | ＞5 | | Reference |  |  |  |  |
|  | ≤5 | | 1.077 (0.714-1.626) |  |  |  |  |
| Differentiation grade | | |  | ＜0.001 |  |  |  |
|  | Well | | Reference |  |  |  |  |
|  | Moderate | | 1.088 (0.631-1.876) |  |  |  |  |
|  | Poor | | 2.072 (1.178-3.645) |  |  |  |  |
| Type of surgery | | |  | 0.846 |  |  |  |
|  | LAR | | Reference |  |  |  |  |
|  | ISR | | 0.913 (0.665-1.235) |  |  |  |  |
|  | APR | | 1.013 (0.640-1.604) |  |  |  |  |
| Surgical approach | | |  | 0.532 |  |  |  |
|  | Robotic surgery | |  |  |  |  |  |
|  | Laparoscopic surgery | | 0.923 (0.721-1.182) |  |  |  |  |
| p T stage | | |  | ＜0.001 |  |  | ＜0.001 |
|  | T1 | | Reference |  |  | Reference |  |
|  | T2 | | 1.066 (0.615-1.848) |  |  | 1.049 (0.604-1.821) |  |
|  | T3 | | 2.437 (1.328-3.255) |  |  | 1.975 (0.889-4.387) |  |
|  | T4 | | 5.658 (3.504-9.137) |  |  | 4.366 (1.921-9.925) |  |
| p N stage | | |  | ＜0.001 |  |  | 0.033 |
|  | N0 | | Reference |  |  | Reference |  |
|  | N1 | | 2.363 (1.784-3.131) |  |  | 1.502 (0.705-3.202) |  |
|  | N2 | | 4.401 (3.234-5.990) |  |  | 2.284 (1.011-5.195) |  |
| Tumor size, mm | | |  | 0.598 |  |  |  |
|  | | ≤40 | Reference |  |  |  |  |
|  | | ＞40 | 0.885 (0.561-1.395) |  |  |  |  |
| Quality of TME | | |  | 0.619 |  |  |  |
|  | | Complete | Reference |  |  |  |  |
|  | | Nearly complete | 1.122 (0.712-1.770) |  |  |  |  |
| R0 resection | | |  | ＜0.001 |  |  | ＜0.001 |
|  | | Yes | Reference |  |  | Reference |  |
|  | | No | 6.112 (4.296-8.670) |  |  | 3.786 (2.581-5.553) |  |
| Neoadjuvant therapy | | |  | 0.076 |  |  |  |
|  | | No | Reference |  |  |  |  |
|  | | Yes | 0.762 (0.564-1.028) |  |  |  |  |
| Adjuvant therapy | | |  | 0.086 |  |  |  |
|  | No | | Reference |  |  |  |  |
|  | Yes | | 0.811 (0.639-1.030) |  |  |  |  |
| HR, hazard ratio; ASA, American society of Aneshesiologists; BMI, body mass index; LAR, low anterior resection; ISR, intersphincteric resection; APR, abdominoperineal resection; TME, total mesorectal excision. | | | | | | | |

**Supplementary Table 8** Univariate and multivariate analyses of risk factors for cancer-specific survival in elderly patients with mid-low rectal cancer after inverse probability of treatment weighting.

| **Variables** | | | **Univariate analysis** | |  | **Multivariate analysis** | |
| --- | --- | --- | --- | --- | --- | --- | --- |
|  |  |  | **HR (95% CI)** | ***p*-value** |  | **HR (95% CI)** | ***p*-value** |
| Age | | |  | 0.134 |  |  |  |
|  | 70-74 | | Reference |  |  |  |  |
|  | 75-79 | | 1.229 (0.859-1.757) |  |  |  |  |
|  | 80+ | | 1.419 (0.933-2.160) |  |  |  |  |
| Sex | | |  | 0.821 |  |  |  |
|  | male | | Reference |  |  |  |  |
|  | female | | 0.964 (0.699-1.328) |  |  |  |  |
| ASA score | | |  | 0.236 |  |  |  |
|  | I or II | | Reference |  |  |  |  |
|  | III or IV | | 1.211 (0.882-1.663) |  |  |  |  |
| Age-adjusted comorbidity index ≤ 2 | | |  | ＜0.001 |  |  |  |
|  | Yes | | Reference |  |  |  |  |
|  | No | | 1.686 (1.238-2.279) |  |  |  |  |
| BMI, kg/m^2^ | | |  | 0.295 |  |  |  |
|  | ≤25 | | Reference |  |  |  |  |
|  | ＞25 | | 1.200 (0.853-1.686) |  |  |  |  |
| History of abdominal surgery | | |  | 0.111 |  |  |  |
|  | No | | Reference |  |  |  |  |
|  | Yes | | 1.423 (0.923-2.196) |  |  |  |  |
| Lateral lymph node dissection | | |  | 0.054 |  |  |  |
|  | No | | Reference |  |  |  |  |
|  | Yes | | 1.575 (0.992-2.499) |  |  |  |  |
| Distance to the anal verge, cm | | |  | 0.837 |  |  |  |
|  | ＞5 | | Reference |  |  |  |  |
|  | ≤5 | | 1.060 (0.609-1.843) |  |  |  |  |
| Differentiation grade | | |  | ＜0.001 |  |  |  |
|  | Well | | Reference |  |  |  |  |
|  | Moderate | | 1.178 (0.609-2.278) |  |  |  |  |
|  | Poor | | 2.492 (1.251-4.962) |  |  |  |  |
| Type of surgery | | |  | 0.814 |  |  |  |
|  | LAR | | Reference |  |  |  |  |
|  | ISR | | 0.957 (0.613-1.494) |  |  |  |  |
|  | APR | | 1.458 (0.837-2.540) |  |  |  |  |
| Surgical approach | | |  | 0.935 |  |  |  |
|  | Robotic surgery | | Reference |  |  |  |  |
|  | Laparoscopic surgery | | 1.013 (0.782-1.298) |  |  |  |  |
| p T stage | | |  | ＜0.001 |  |  | ＜0.001 |
|  | T1 | | Reference |  |  | Reference |  |
|  | T2 | | 0.751 (0.372-1.513) |  |  | 0.726 (0.356-1.479) |  |
|  | T3 | | 1.736 (0.972-3.100) |  |  | 1.375 (0.754-2.507) |  |
|  | T4 | | 5.047 (2.712-9.392) |  |  | 3.131 (1.577-6.220) |  |
| p N stage | | |  | ＜0.001 |  |  | ＜0.001 |
|  | N0 | | Reference |  |  | Reference |  |
|  | N1 | | 1.721 (1.179-2.514) |  |  | 1.406 (0.959-2.059) |  |
|  | N2 | | 4.440 (3.062-6.438) |  |  | 2.647 (1.709-4.098) |  |
| Tumor size, mm | | |  | 0.714 |  |  |  |
|  | | ≤40 | Reference |  |  |  |  |
|  | | ＞40 | 1.077 (0.725-1.598) |  |  |  |  |
| Quality of TME | | |  | 0.869 |  |  |  |
|  | | Reference | Reference |  |  |  |  |
|  | | Nearly complete | 0.956 (0.560-1.633) |  |  |  |  |
| R0 resection | | |  | ＜0.001 |  |  | ＜0.001 |
|  | | Yes | Reference |  |  | Reference |  |
|  | | No | 4.424 (2.929-6.685) |  |  | 3.512 (2.253-5.474) |  |
| Neoadjuvant therapy | | |  | 0.480 |  |  |  |
|  | | No | Reference |  |  |  |  |
|  | | Yes | 0.799 (0.428-1.492) |  |  |  |  |
| Adjuvant therapy | | |  | 0.173 |  |  |  |
|  | No | | Reference |  |  |  |  |
|  | Yes | | 0.805 (0.590-1.100) |  |  |  |  |
| HR, hazard ratio; ASA, American society of Aneshesiologists; BMI, body mass index; LAR, low anterior resection; ISR, intersphincteric resection; APR, abdominoperineal resection; TME, total mesorectal excision. | | | | | | | |
